# p53 dynamics in single cells are temperature-sensitive

**DOI:** 10.1038/s41598-020-58267-1

**Published:** 2020-01-30

**Authors:** Marcel Jentsch, Petra Snyder, Caibin Sheng, Elena Cristiano, Alexander Loewer

**Affiliations:** 1 Department of Biology, Technische Universität Darmstadt, Darmstadt, Germany; 2 Present Address: Novartis Institutes for Biomedical Research, Oncology Disease Area, Basel, Switzerland

**Keywords:** Tumour-suppressor proteins, Data processing, Oscillators, Single-cell imaging

## Abstract

Cells need to preserve genome integrity despite varying cellular and physical states. p53, the guardian of the genome, plays a crucial role in the cellular response to DNA damage by triggering cell cycle arrest, apoptosis or senescence. Mutations in p53 or alterations in its regulatory network are major driving forces in tumorigenesis. As multiple studies indicate beneficial effects for hyperthermic treatments during radiation- or chemotherapy of human cancers, we aimed to understand how p53 dynamics after genotoxic stress are modulated by changes in temperature across a physiological relevant range. To this end, we employed a combination of time-resolved live-cell microscopy and computational analysis techniques to characterise the p53 response in thousands of individual cells. Our results demonstrate that p53 dynamics upon ionizing radiation are temperature dependent. In the range of 33 °C to 39 °C, pulsatile p53 dynamics are modulated in their frequency. Above 40 °C, which corresponds to mild hyperthermia in a clinical setting, we observed a reversible phase transition towards sustained hyperaccumulation of p53 disrupting its canonical response to DNA double strand breaks. Moreover, we provide evidence that mild hyperthermia alone is sufficient to induce a p53 response in the absence of genotoxic stress. These insights highlight how the p53-mediated DNA damage response is affected by alterations in the physical state of a cell and how this can be exploited by appropriate timing of combination therapies to increase the efficiency of cancer treatments.

## Introduction

Preserving genome integrity is a central function of all metazoan cells. Therefore, complex mechanisms evolved to detect and respond to DNA damage. However, the underlying molecular networks are subjected to varying physical and cellular states that limit their functionality. Among the most basic physical properties that influence molecular functions is temperature. Previous studied revealed that some cellular networks such as circadian clocks are compensated towards changes in temperatures^1^, while others such as the NF-kB-mediated response to inflammatory cytokines show temperature-dependent changes in their dynamics^2^. To determine to which extent temperature affects the cellular capacity to preserve genome integrity, we focused on the molecular network controlling the tumour suppressor p53.

The transcription factor p53 is activated by cellular stresses such as DNA damage and activates the expression of downstream genes that mediate cellular responses ranging from transient cell cycle arrest and damage repair to terminal cell fates such as senescence or apoptosis^3,4^. In unstressed cells, p53 protein is kept at low levels by Mdm2 - mediated ubiquitination and subsequent proteasomal degradation^5^. Posttranslational modifications by DNA damage-activated kinases such as ATM and ATR disrupt this interaction and lead to p53 stabilization and accumulation in the nucleus. P53-mediated signalling is a highly dynamic process that integrates information from various sources to regulate the cellular stress response^6,7^. Therefore, the temporal pattern of p53 accumulation correlates with the type and quantity of the stimulus^8^. Ionizing radiation (IR) induces uniform pulses of p53 accumulation with comparable amplitude and duration^9,10^. The number of pulses is positively correlated with the damage dose^11^. These pulsatile p53 dynamics are triggered by negative feedback loops within the p53 signalling network, mainly through the ubiquitin ligases Mdm2 and the phosphatase PPM1D/Wip1^9,12,13^. In contrast, p53 exhibits only a single sustained pulse with dose-dependent amplitude and duration in response to ultraviolet (UV) radiation^8^. Using pharmacological and genetic perturbation, we have previously shown that p53 dynamics contribute to cell fate choices post damage^14,15^. In most human cancers, p53 is inactivated either directly by mutations that compromise the stability of the protein or its ability to bind DNA, or indirectly by amplifications of its negative regulators such as Mdm2 or Wip1^16^. Inactivation of p53 correlates with increased resistance to therapy and poor survival^17^.

As homoeothermic organisms, humans maintain a stable core body temperature of about 37 °C at rest. However, body temperature at the periphery and specifically at the extremities drops significantly to 33 °C or lower, depending on ambient temperatures^18,19^. Other factors such as age, time of day, sex, reproductive status and activity level lead to deviating body temperatures as well^20,21^. Moreover, during physical exertion, the physiological range of core temperatures extends beyond 40 °C^22^. Even higher core temperatures are observed in pathological settings such as vascular disease where the transport of blood and heat is perturbed, or during fever, which is associated with several different diseases including infections, autoimmune diseases^23^ and cancer^24^.

Changes in temperature affect organisms on multiple levels. Its direct impact on enzyme-catalysed reactions is characterized by the Arrhenius equation, which describes that reaction velocities double when temperature increases by 10 °C until they abruptly decline due to protein denaturation^25^. On the cellular level, temperature changes affect metabolism, protein synthesis and cell cycle regulation^26^. Hyperthermia, in particular, causes membrane, DNA and protein damage and influences cell fate by triggering cell cycle arrest, senescence, apoptosis, or necrotic cell death^27–31^. On the organismic level, studies indicate that deviations in core temperature of more than 4 °C can result in physiological impairments and fatality^32^.

Previous studies investigating the effect of temperature changes on the DNA damage response (DDR) mainly focused on the damage-activated kinases ATM and ATR and their downstream effectors Chk1 and Chk2, which orchestrate a network of cellular processes to maintain genomic integrity^33^. It has been observed that hyperthermia leads to activation of human ATR inducing cell-cycle arrest at the G2/M transition^34^. Furthermore, activated ATR may help to stabilize arrested replication forks preventing accumulation of chromosomal lesions^35^. Similarly, hyperthermia induces auto-phosphorylation and activation of ATM^36^, which is however delayed by 1–2 hours compared to ATR activation^34^. Interestingly, other components of the DDR are inhibited at high temperatures: the Mre11-Rad50-Nbs1 (MRN) complex, which is essential for ATM activation by DNA double strand breaks (DSBs), translocates from the nucleus to the cytosol at high temperatures^37^ and the binding of 53BP1 to chromatin around DSBs is prevented^36,38^, restricting the capacity of ATM to sense this form of DNA damage.

Clinically, the application of exogenous heat in combination with canonical anti-cancer treatments is an established therapeutic strategy against various human tumours^39^. Several randomized trials have demonstrated the benefits of hyperthermia (40–42 °C for at least 1 hour) combined with radiotherapy or chemotherapy with regard to local tumour control and survival^40–42^. Hyperthermia also amplifies the therapeutic effect of chemotherapy^43–46^. Pre-clinical and clinical studies suggest that hyperthermia is effective both in cancers with wild-type p53 and those without active p53. To our knowledge, the p53 status of cancers being treated with hyperthermia is not routinely determined in the clinic^47,48^. Molecularly, it was shown that cancer cells get arrested in different stages of the cell cycle (S/G2 or G2/M) and lose the ability to repair chromosomal lesions, which promotes cell death^34,49–51^.

In response to temperature changes, a family of heat shock factors (HSF1-4) initiates the expression of heat shock proteins (HSP). The most well-known family member is HSF1, which is constitutively expressed and regulates the expression of HSP as well as other genes related to cell signalling, cell integrity, development, growth, fertility, senescence and apoptosis^52–54^. HSF1 has been reported to interact at different levels with the p53 network by supporting post-translational modifications and acting as a transcriptional co-factor^55^. Furthermore, interaction of the HSF1 target Hsp90 with p53 prevents Mdm2 mediated ubiquitination^56^ and modulates its binding to the CDKN1A/p21 promotor^55^. Additional heat shock proteins such as Hsp70 and Hsp40 are as well capable of stabilizing p53-DNA complexes^57,58^.

We now aimed to systematically study how temperature affects p53 signalling. Is the molecular network controlling activation of the main human tumour suppressor compensated against changes in temperature or does its response to DNA damage dependent on the physical environment? To address this question, we employed an established fluorescent reporter cell line^59^ and followed the dynamics of p53 accumulation after ionizing radiation at the single cell level using time-resolved live-cell microscopy. Computer-aided image and data analysis indicated that the dynamic features of the pulsatile p53 response vary with temperature within the range of 33 °C to 39 °C. Above 40 °C, we observed a reversible phase transition to fundamentally different p53 accumulation dynamics, which may provide new insights how hyperthermia can be used to improve cancer therapy.

## Results

### P53 dynamics upon genotoxic stress are temperature dependent

**Figure 1:**
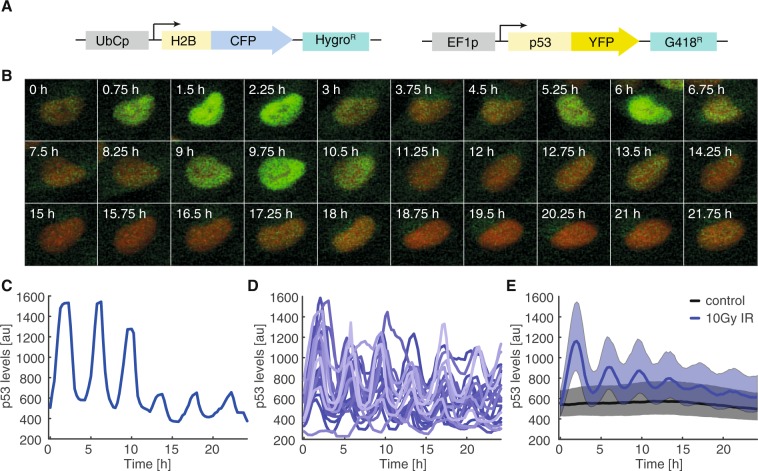
p53 dynamics in individual cells (**A**) Schematic overview of the reporter system used to monitor p53 dynamics. P53 fused to the yellow fluorescent protein mVenus (YFP) is stably expressed under control of the EF1A promoter. H2B is fused to mCerulean (CFP) to track nuclei in time-lapse microscopy images. (**B**) Time-lapse microscopy images of a selected cell irradiated with 10 Gy. The marker H2B-CFP is shown in red, p53-YFP in green. (**C**) Median nuclear p53-YFP levels were quantified over time for the cell shown in (B). (**D**) P53 levels were quantified in thousands of cells. To demonstrate the heterogeneity of the response, 20 randomly picked trajectories of p53 levels after 10 Gy ionizing radiation were visualized. (**E**) The p53 response on the population level at 37° (black: non-irradiated control cells; blue: cells irradiated with 10 Gy). Bold lines indicate median p53 levels, shaded areas reflect the inter-quantile range between the 0.25 and 0.75 quantile. See Table S1 for number of analysed cells.

To monitor p53 dynamics, we employed a clonal A549 lung carcinoma cell line stably expressing p53 fused to the yellow fluorescent protein mVenus and the nuclear marker H2B fused to a cyan fluorescent protein (mCerulean) (Fig. 1A)^59^. We irradiated cells with 10 Gy X-ray (250 kV) and monitored their p53 protein levels for 24 h by time-resolved live-cell microscopy. As previously described, p53 protein accumulated in a pulsatile manner upon damage induction (Fig. 1B)^8^. Using computer-aided image analysis, we followed thousands of individual cells and quantified their p53 response (see methods sections for details). Trajectories of individual cells confirmed uniform pulsatile p53 dynamics upon damage induction (Fig. 1C) that were asynchronous and heterogeneous across the population (Fig. 1D). This led to the appearance of damped pulses in the median p53 level of cell populations (Fig. 1E)^60^.

**Figure 2:**
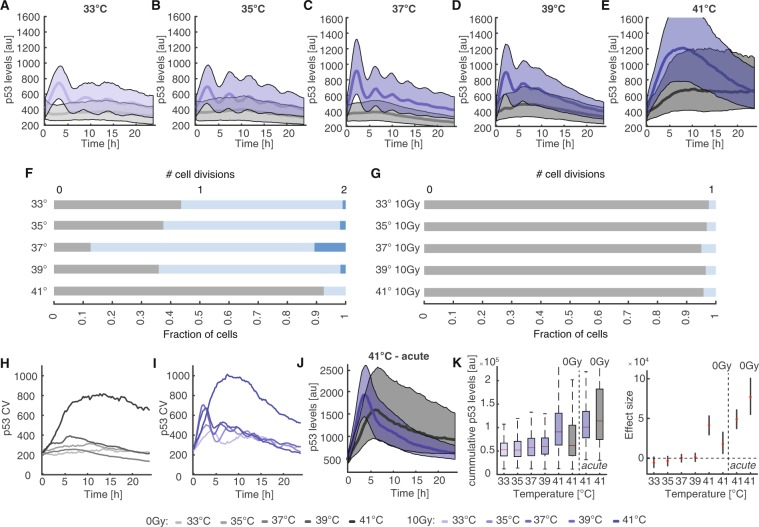
p53 dynamics are temperature sensitive (**A–E**) Population level p53 responses of pre-equilibrated cells for the temperature range from 33 °C to 41 °C (black: non-irradiated, blue: 10 Gy IR). Bold lines indicate median p53 levels, shaded areas reflect the inter-quantile ranges from 0.25 to 0.75. At 41 °C, p53 dynamics change dramatically, as pulsatile p53 accumulation gets supplanted by a strong sustained response. See Table S1 for number of analysed cells. (**F–G**) Cell proliferation shown as number of cell divisions per cell within 24 h for non-irradiated (**F**) and irradiated (**G**) cells incubated at the indicated temperatures. (**H–I**) Coefficients of variation over time for control (**H**) or damaged (**I**) cell populations shown in (**A**–**E**). At 41 °C, we detected strong increases in heterogeneity for both conditions. At other temperatures heterogeneity was mainly caused by increasing asynchrony of p53 pulses within a population. (**J**) p53 response of control and irradiated cell populations acutely shifted to 41 °C without prior pre-equilibration. The responses differ in amplitude and dynamics from our observations in (**E**). See Table S1 for number of analysed cells. (**K**) Accumulated p53 levels for non-irradiated and irradiated cells over the temperature range from 33 °C to 41 °C for the observation period of 24 h (left). Estimated changes compared to cells incubated at 37 °C are indicated on the right (red dots); error bars represent 95% confidence intervals determined by permutation testing. Dashed lines serve as guides to the eye.

To investigate the influence of varying temperature on the p53 response to genotoxic stress, we equilibrated cells to temperatures ranging from 33 °C to 41 °C for 14 h and performed time-lapse microscopy of untreated and irradiated cells at the respective temperatures (Fig. 2A–E). To compare p53 dynamics from different microscopy experiments, we normalized the resulting data under the assumption of equal initial p53 distributions (Supp. Fig. S1, see methods for details). When lowering temperature, we monitored an increased duration of p53 pulses and a reduced frequency in the median trajectories of corresponding populations (Fig. 2A–B) compared to cells damaged at 37 °C (Fig. 2C, Supp. Fig. S2A,B and Supp. Movies S1–6). Untreated cells did not show noticeable changes in p53 levels at 33 °C or 35 °C (Supp. Fig. S2E,F). When we increased the temperature to 39 °C, we could observe the opposite effect: pulse duration decreased and pulses appeared to happen faster (Fig. 2D, Supp. Fig. S2C and Supp. Movies 7 and 8). Interestingly, even untreated cells showed minor increases in p53 levels during time-lapse microscopy (Fig. 2D, black line/shaded area and Supp. Fig. S2G). Finally, when we increased temperature to 41 °C, which corresponds to mild hyperthermia in a clinical setting, we found a striking change in p53 dynamics: instead of regular pulses of p53 accumulation, we now observed a single sustained p53 pulse of long duration after damage induction (Fig. 2E, Supp. Fig. S2D,I and Supp. Movies S9 and 10). The maximum increase in the p53 level was about 1.5 fold higher than the average amplitude of the first pulse at 37 °C. Even in the absence of damage induction, p53 levels strongly accumulated in the nucleus during the observation period (Fig. 2E, black line/shaded area and Supp. Fig. 2H,I), reaching around 60% of the average amplitude of the first pulse at 37 °C.

When we analysed how untreated cells proliferated at different temperatures, we observed decreased proliferation rates below and above normothermia as expected (Fig. 2F). At 41 °C, most cells stopped dividing. Upon irradiation, cells remained arrested for the duration of the experiment at all temperatures tested (Fig. 2G). In addition to changes in dynamics and proliferation, we also observed a strong increase in heterogeneity when moving from 39 °C to 41 °C both in treated and untreated cells (Fig. 2H,I). Similar results were observed in the non-transformed diploid breast epithelial cell line MCF10A expressing p53 fused to mVenus from the endogenous gene locus (Supp. Fig. S3)^61^. Interestingly, MCF10A cells tended to show sustained p53 accumulation already at 39 °C.

As we were surprised by the strong response of untreated cells at 41 °C despite equilibrating cells to the higher temperature, we compared the p53 response in cells pre-equilibrated to 41 °C with cells that were acutely shifted to the higher temperature at the beginning of the experiment. (Fig. 2J). In pre-equilibrated cells, we observed a fast and strong increase of the nuclear p53 level upon damage, reaching a maximum around 8 h after the stimulus. Afterwards, p53 levels declined until the end of the observation period (Fig. 2E). p53 levels in non-irradiated cells increased more slowly and reached a plateau at about 60% of the peak p53 levels after irradiation. In cells directly exposed to 41 °C, nuclear p53 accumulated faster upon damage induction, reaching peak-levels already 4 h post irradiation. P53 levels than decayed quickly during the remaining 20 h (Fig. 2J). Non-irradiated cells showed a similarly strong increase upon the temperature shift, reaching around 85% of peak levels in damaged cells after 6 h. Interestingly, p53 levels then declined slower than in irradiated cells, which led to on average higher p53 levels in non-treated cells compared to damaged cells (Fig. 2J).

To further compare the strength of the p53 response at different temperatures and treatment conditions, we quantified the integrated nuclear level of p53 for the observation period. Over the temperature range from 33 °C to 39 °C, cumulative p53 levels remained similar in undamaged cells and upon irradiation (Fig. 2K). At 41 °C, median integrated p53 levels of non-irradiated pre-equilibrated cells were comparable to those of damaged cells at lower temperature, although we observed a wider distribution across the population. Cells irradiated at 41 °C showed similar cumulative p53 levels, no matter whether they were pre-equilibrated or acutely shifted to the higher temperature, despite diverging dynamics. Strikingly, non-irradiated cells acutely exposed to 41 °C showed on average the strongest p53 response. The differences between cells pre-equilibrated at or directly exposed to 41 °C point to a decreased sensitivity to hyperthermia of pre-equilibrated cells.

### Pulsatile p53 dynamics are disrupted at 41 °C

**Figure 3:**
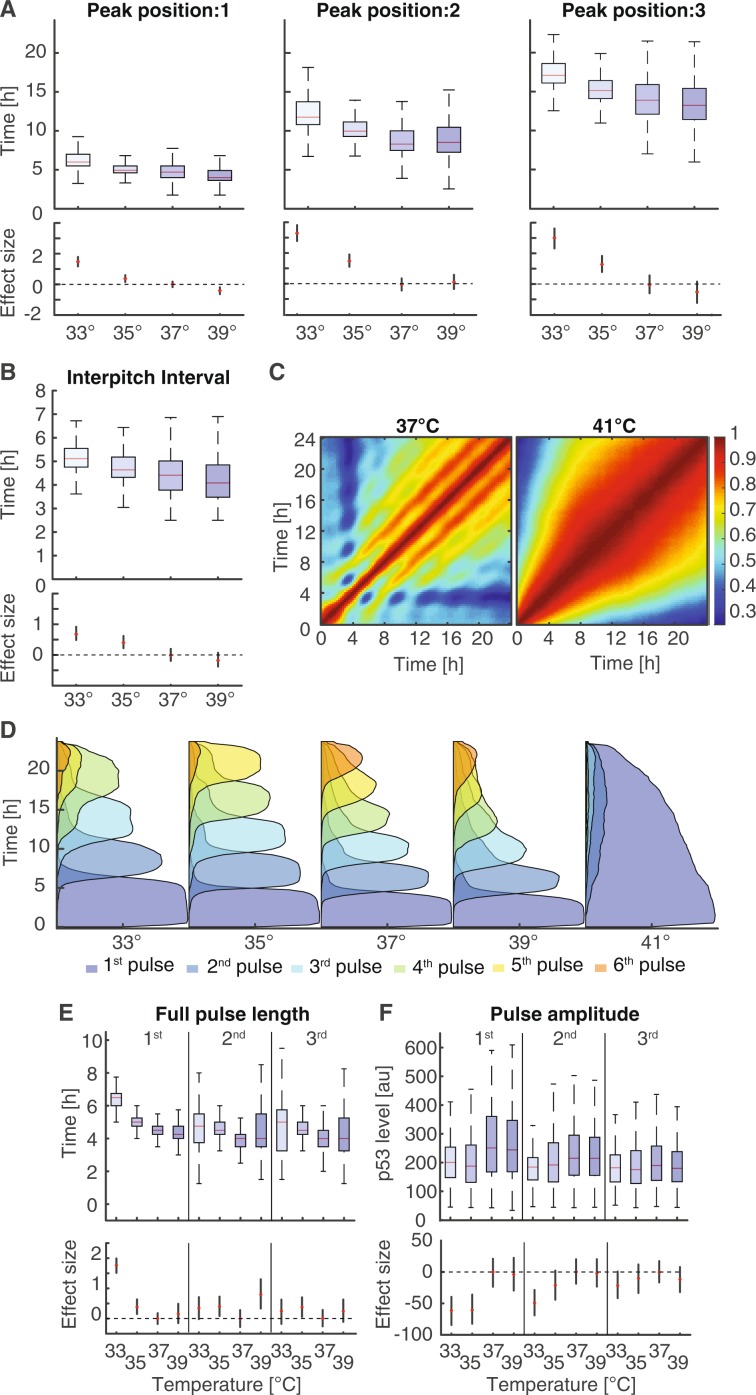
Quantification of dynamic features of the p53 response (**A**) Quantification of the first 3 pitch positions estimated by the average magnitude difference function for irradiated cells (10 Gy) over a temperature range from 33 °C to 37 °C. Red lines indicate medians of distributions; boxes include data between the 25th and 75th percentiles; whiskers extend to maximum values within 1.5 × the interquartile range. Estimated changes compared to cells incubated at 37 °C are indicated below (red dots); error bars represent 95% confidence intervals determined by permutation testing. Dashed lines serve as guides to the eye. (**B**) Quantification of the inter-pitch intervals for irradiated cells (10 Gy) over a temperature range from 33 °C to 37 °C. With lower temperatures the length of the interval increases while the range of the distribution is reduced. Estimated changes compared to cells incubated at 37 °C are indicated below (red dots); error bars represent 95% confidence intervals determined by permutation testing. Dashed lines serve as guides to the eye. (**C**) Auto-correlation among irradiated cells at 37 °C and 41 °C. Each point in this graph represents the correlation coefficient between p53 levels in the population at two different time points, except along the main-diagonal. We detected repetitive pulses for 37 °C, but only one long homogenous response for 41 °C. (**D**) Probabilities of being in a certain pulse state at a given time point for irradiated cells (10 Gy) over a temperature range from 33 °C to 41 °C. Pulses are detected using a local dynamic time warping based approach. The pulsing frequency is positively correlated with the temperature within the range between 33 °C to 39 °C. At 41 °C the pulsing dynamics is completely lost and replaced by a sustained response. (**E–F**) Quantification the duration (**E**) and amplitude (**F**) of the first 3 pulses with respect to temperature. Estimated changes compared to cells incubated at 37 °C are indicated below (red dots); error bars represent 95% confidence intervals determined by permutation testing. Dashed lines serve as guides to the eye.

To get a better understanding of the temperature dependence of p53 dynamics at the single cell level, we employed the Average Magnitude Difference Function (AMDF)^62^ to estimate the pitch of p53 pulses in single cell trajectories. The positions of the first 3 pulses varied substantially between the different temperatures (Fig. 3A). At 33 °C, the first p53 peak occurred on average 1.5 h later than at 37 °C. Conversely, cells at 39 °C responded on average faster than cells at 37 °C, although distributions of first pitch positions overlapped in the range from 35 °C to 39 °C. We measured the same trend for the 2^nd^ and 3^rd^ pulse (Fig. 3A). Furthermore, we observed an increase in the variability of the pitch position with increasing temperature. Temperature-dependent changes in pulse timing and synchrony led to decreasing median inter-pulse intervals at increasing temperature while the width of the corresponding distributions increased (Fig. 3B). An alternative way to visualize pulsatile p53 dynamics is to plot auto-correlation coefficients of nuclear p53 levels at different time points across populations of cells. The resulting heatmaps clearly show temperature-dependent changes of the regular p53 accumulation in the range from 33 °C to 39 °C (Fig. 3C, left and Supp. Fig. 4). In contrast, no periodicity was observable in cells damaged at 41 °C (Fig. 3C, right). Instead, the p53 response was correlated over extended periods of times, indicating that cells with a strong immediate response also reacted more strongly at later time points compared to cells with a weaker early response.

A good measure to quantify temperature sensitivity is the Q_10_ temperature coefficient^1^, which reflects the rate of change of biological systems when temperature is raised by 10 °C assuming that reaction rates depend exponentially on temperature. For the p53 system, we determined a Q_10_ coefficient of 1.53 ± 0.24 based on the position of the 3^rd^ pitch, which is on the lower end of coefficients usually observed in biology (Q_10_ ~ 2-3)^63^.

As autocorrelation-based pitch detection is designed to recognize repeating patterns across the entire trajectory, we employed a feature detection approach based on local dynamic time warping^64^ inspired by the Smith-Waterman algorithm^65^ to characterized individual p53 pulses (Supp. Fig. S5A–C, see methods for details). We first determined probabilities for detecting a certain feature at a given time point in individual trajectories and again observed temperature-dependent changes in timing from 33 °C to 39 °C as expected (Fig. 3D and Supp. Fig. S5D–G). At 41 °C, pulsatile dynamics broke down and we observed only one extended feature in most cells (Fig. 3D and Supp. S5H). In addition to changes in pulse frequency, this analysis also confirmed changes in synchrony of features with temperature. We observed a similar dependency of dynamic features on temperature in MCF10A cells, although they showed widespread break down of pulsatile dynamics already at 39 °C (Supp. Fig. S5I). We next analysed the length of the first three pulses in A549 cells. The length of the first pulse increased from about 4.5 h to 6.5 h as the temperature was lowered from 37 °C to 33 °C (Fig. 3E). Subsequent pulses are in general shorter, however, median durations again increased with lower temperatures. At 39 °C, the average length of later pulses was similar to 37 °C, although the distributions were markedly wider. Pulse amplitudes defined as the difference between maximal and minimal p53 levels within a pulse were damped for the first pulse at temperatures below 37 °C (Fig. 3F). At 39 °C, they remained equal to normothermia. Interestingly, these differences diminished for later pulses.

As previous reports have shown that p53 dynamics affect the cellular outcome of the DNA damage response^14,66^, we aimed to determine if the expression of p53 target genes upon damage induction is temperature-dependent as well. To this end, we focused on a set of high-confidence target genes that contribute to distinct cellular response pathways, including CDKN1A/p21, GADD45 (both cell cycle arrest), XPC (DNA damage repair) and BAX (apoptosis), and measured corresponding RNA levels at different time points and temperatures in untreated and irradiated cells by qPCR^3^. For p21, GADD45 and, to a lesser extent, XPC, we observed increased expression at lower temperatures (35 °C) when p53 showed longer and less frequent accumulation pulses (Supp. Fig. S6A–D). However, these target genes were also affected by temperature shifts alone, indicating a combined effect of altered p53 dynamics and temperature-induced changes in gene expression. Interestingly, we observed increased expression of BAX at higher temperatures (39 °C) at late time points post damage, suggesting an increased tendency to induce apoptosis under these conditions. At 41 °C, expression of CDKN1A and GADD45 was attenuated, while expression of BAX and XPC was slightly increased, following the trends observed at 39 °C (Supp. Fig. S6E–H). We also detected less cells arrested in G2 24 h and 48 h after irradiation at 41 °C as well as a slight increase in apoptotic cells (Supp. Fig S6I,J).

In MCF10A cells, which express p21 protein tagged with the red fluorescent protein mCherry from the endogenous locus as well, we similarly observed that the immediate p21 response at about 8 h was strongest at 33 °C and decreased in amplitude with increasing temperature (Supp. Fig. S3E–H). Interestingly, the sustained accumulation of p21 at later time periods between 12 h and 24 h was most pronounced at 37 °C.

### An ongoing p53 response is modulated by changes in temperature

**Figure 4:**
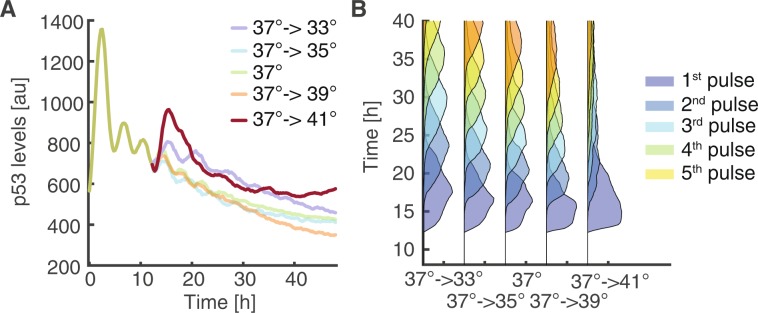
An ongoing p53 response is altered by changes in temperature (**A**) Temperature change alters the dynamics of an ongoing p53 response. Cells were incubated at 37 °C and exposed to genotoxic stress (10 Gy IR). 12 h after damage induction, approximately after the 3^rd^ pulse of the expected canonical response, we altered the temperature in the range from 33 °C to 41 °C and followed cells for an additional 36 h. Median p53 levels of cell populations are indicated. See Table S1 for number of analysed cells. (**B**) Probabilities of being in a certain pulse after temperature change at 12 h.

As we observed a strong temperature-dependency of p53 dynamics when we irradiated cells at different temperatures, we next investigated how dynamic features are affected by temperature changes during an ongoing response to genotoxic stress. To this end, we equilibrated cells at 37 °C, performed time-lapse microscopy upon irradiation with 10 Gy X-ray and altered temperature in a range from 33 to 41 °C at 12 h approximately after the 3^rd^ p53 pulse (Fig. 4A). Even though p53 was already accumulating in a regular pulsatile manner, lowering temperature led to a reduced frequency of p53 pulses, as indicated by feature analysis starting from the time of temperature shifts (Fig. 4B). Correspondingly, increasing temperature to 39 °C increased pulse frequency as well as heterogeneity. Shifting cells to 41 °C induced a strong immediate but transient p53 response without further pulsing.

**Figure 5:**
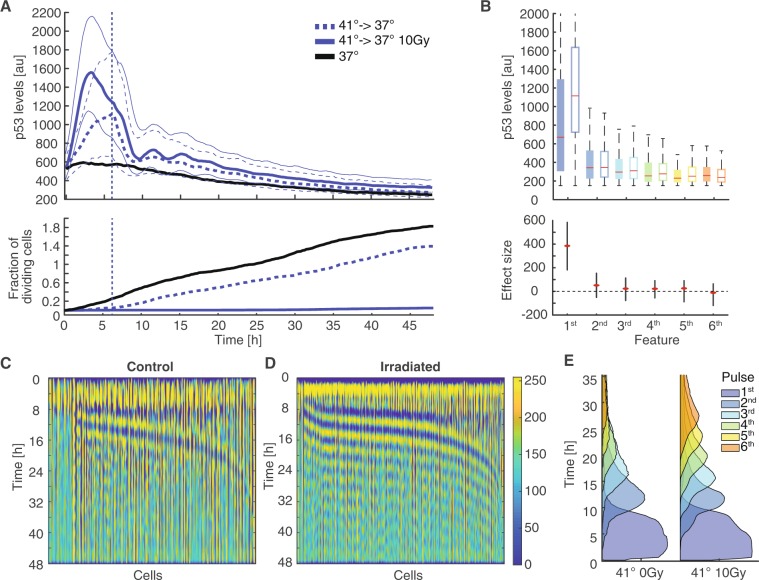
The p53 response to hyperthermia is reversible (**A**) Cells pre-equilibrated at 41 °C (see Fig. 2) were tracked for 48 h. After 6 h, temperature was reduced to 37 °C. We observed immediate decreases in p53 levels followed by pulsatile dynamics. Median p53 levels of cell populations are indicated. See Table S1 for number of analysed cells. The lower graph indicates corresponding cumulative fractions of cells dividing over time. (**B**) Quantification of amplitudes for the first 6 pulses detected by the dynamic time warping approach for cell populations shown in (A). The first feature corresponds to the acute p53 response to hyperthermia. Subsequent pulses mainly detected after reducing the temperature do not differ among irradiated (open boxes) and non-irradiated cells (solid boxes). Red lines indicate medians of distributions; boxes include data between the 25th and 75th percentiles; whiskers extend to maximum values within 1.5 × the interquartile range. Estimated changes between irradiated and non-irradiated cells are indicated below (red dots); error bars represent 95% confidence intervals determined by permutation testing. Dashed lines serve as guides to the eye. (**C–D**) Heatmap of normalized p53 levels for the non-irradiated (**C**) and irradiated (**D**) cell population sorted by the position of the first new feature appearing after the temperature was set to 37 °C (6 h). The data shown was adjusted using images processing techniques to enhance visualization of pulsatile dynamics (see methods section). (**E**) Probabilities of being in a certain feature at a given time point for irradiated and non-irradiated cells. The induced pulsing is less heterogeneous for irradiated cells. Non-irradiated cells have on average less pulses.

The observed immediate effects of temperatures changes on p53 dynamics prompted us to further investigate p53 induction by hyperthermia alone. We were specifically puzzled by the increased p53 levels observed in non-damaged cells pre-equilibrated to 41 °C. We hypothesized that short-term temperature changes during irradiation were sufficient to reset the state of the p53 system by inducing re-folding of the protein, reverting potential interactions with HSPs and inducing re-expression of Mdm2. To test this hypothesis, we equilibrated cells to a temperature of 41 °C and performed time-lapse microscopy of untreated and irradiated cells. The temperature was kept at 41 °C for the first 6 h and afterwards decreased to normothermia at 37 °C. This drop in temperature took around 30 minutes due to the technical capabilities of our incubation system. As previously observed, irradiated and untreated cells responded with a strong initial p53 response to hyperthermia conditions. However, as soon as the temperature change back to normothermia was initiated, p53 levels decreased and irradiated cells started to show pulsatile dynamics at about 9 h post damage (Fig. 5A, upper graph). Surprisingly, we also detected pulses in non-irradiated cells after the p53 response to hyperthermia decayed. Non-treated cells stopped proliferation at 41 °C (Fig. 5A, lower graph). However, they continued to divide soon after the return to normothermia with similar rates as cells incubated continuously at 37 °C. Irradiated cells remained arrested for 48 h independent of the temperature.

To quantify the observed pulses after transient temperature shifts, we applied our feature detection approach. As expected, the immediate response to hyperthermia was similar in amplitude to previously observations. Interestingly, the later p53 accumulation pulses were of comparable amplitude in both irradiated and undamaged cells (Fig. 5B). However, the timing and synchrony of p53 pulses differed substantially (Fig. 5C–E). In irradiated cells, we observed similar relatively homogeneous dynamic patterns as in irradiated cells constantly incubated at 37 °C. In contrast, the p53 response induced by hyperthermia alone was more heterogeneous, with delayed and asynchronous occurrence of p53 peaks, arguing for a different molecular mechanism controlling p53 accumulation in the absence of radiation-induced kinase activity.

## Discussion

Reliable information processing by the p53 pathway is crucial to ensure an appropriate cellular response to genotoxic stress in a dynamically changing environment. Perturbation of p53 signalling leads to transformation of individual cells and subsequent tumorigenesis. To understand the influence of varying temperature on the dynamics of the p53 network upon ionizing radiation, we monitored p53 protein levels of thousands of genetically identical cells exposed to the physiologically relevant temperature range of 33 °C to 41 °C. Using a combined computational and experimental single cell approach we have shown that the frequency of p53 accumulation pulses that encode DNA damage is positively correlated with temperatures up to 39 °C. Interestingly, heterogeneity of p53 dynamics increased with temperature as well. These results highlight that despite its importance for maintaining genome stability, the p53 network is not temperature-compensated at the level of p53 accumulation dynamics. Similar observations have been recently reported for the NF-kB network^2^. Here, information is encoded in oscillations of cytoplasmic to nuclear translocation of the transcription factor RelA, which also increase in frequency with temperature. It was shown that temperature-dependent pathway dynamics are caused by differential timing of feedback expression and affected only a subset of target genes at early time points. Further investigations will show if similar principles are applicable to p53 signalling as well or if the observed changes reflect an integrated property of the entire network. Interestingly, we also observed that temperature dependent changes in target gene expression were mainly restricted to early time points after damage induction, which may suggest that over longer time periods, temperature compensation is achieved at the level of target genes.

To our surprise, we observed a phase transition at 41 °C from canonical pulsatile dynamic to a strong initial response followed by sustained nuclear p53 levels, which was reversible upon return to normothermia. A similar p53 response was detected in non-irradiated cells exposed to mild hyperthermia. Interestingly, reversion of non-irradiated cells to normothermia induced heterogeneous but sustained p53 pulsing for several hours. The molecular mechanism of this phase transition is so far unknown. We speculate that it is based on structural properties of p53 and Mdm2 as well as their interaction with HSPs. It is well documented that p53 folding is marginally stable at physiological temperature. This is highlighted by cancer-related p53 mutants that often show temperature-sensitive phenotypes^67,68^. Unfolding of a substantial fraction of p53 molecules at 41 °C could prevent their interaction with Mdm2 and subsequent proteasomal degradation. Unfolded p53 would be transcriptionally inactive, breaking the feedback loop that restricts the duration of the p53 response under normothermic conditions. Furthermore, increased levels of Hsp90 upon hyperthermia may contribute to p53 stabilization, as the chaperon stabilize p53 conformations that oppose Mdm2 binding, and aids Mdm2 degradation^57,69,70^. Interestingly, cells that were pre-incubated at 41 °C showed a reduced p53 response to hyperthermia, indicating that p53 signalling acquires tolerance to heat shocks. The underlying mechanisms and the role of the HSF-HSP system will have to be clarified in further studies.

What are the physiological consequences of the observed temperature-dependent p53 dynamics upon genotoxic stress? Due to its central importance for guarding genomic integrity, appropriate p53 function must be ensured across physiological temperature ranges. Prolonged p53 accumulation pulses during hypothermia may contribute to counteract generally decreased synthesis rates and ensure sufficient expression of crucial target genes such as p21. In this way, p53 function rather than its dynamics would be temperature compensated, similar to the NF-kB system^2^. Sustained p53 accumulation during hyperthermia may serve a different purpose. It is well known that hyperthermia leads to mitotic phenotypes and genomic instability^71^ as well as an attenuated damage response^35^. Sustained but reversible p53 accumulation may therefore ensure that cells induce transient cell cycle arrest during hyperthermia and only enter mitosis if physical conditions are permissive.

In the last decades, hyperthermia in combination with radio- and/or chemotherapy has proven to be beneficial for the treatment of human cancers and has gained particular attention due to negligible side effects^72,73^. How hyperthermia influences the outcome of cancer treatment on the molecular level by modulating cellular and molecular pathways is not well examined and remains an open question^47^. Some studies using tissue culture and animal models suggest that wild-type p53 may positively affect the efficiency of combined treatments with radiation and hyperthermia. In addition, some clinical studies indicate that the p53 status of a tumour may be an important predictor for the success of hyperthermia-based therapies^48,74^. Other studies indicate that apoptosis rate rather than p53 status is the better predictor of clinical outcomes^47,75^. In this regard, the molecular insights obtained in our study remain inconclusive: while transient induction of cell cycle arrest protects actively cycling cells from entering mitosis at non-permissive temperatures, which would otherwise lead to effective cell killing through mitotic catastrophe, the observed hyperaccumulation at temperatures above 41 °C degrees suggests that p53 wild-type cancer cells may be susceptible to increased induction of apoptosis or senescence under these conditions. This would be specifically important in situations where p53 function is disabled through amplification of its negative regulator Mdm2, as often observed in sarcomas^76,77^. Moreover, the diverse functions of Hsp90 in the p53 network may contribute to positive effects in cancer treatment: Hsp90 accumulated during hyperthermia may promote refolding of destabilized mutant p53 upon return to normothermia, compensating functional deficiencies in cancer cells. It will be interesting to determine the dynamics of mutant p53 in response to irradiation at different temperatures in future studies and systematically compare the outcome of combined treatments in cells with and without active p53. In any case, the observed immediate and reversible effects of hyperthermia on p53 dynamics as well as the adaptation of the p53 signalling network to heat exposure provide molecular evidence that the precise timing of radiation and hyperthermic treatments is a critical parameter for successful combination therapies^78,79^. Further studies using our combined computational and experimental single cell approach will allow us to gain a deeper understanding of how varying physical and cellular states affect the function of the p53 network and how we can exploit this to devise more efficient tumour therapies.

## Material and Methods

### Cells

The human lung carcinoma cell line A549 expressing p53-mVenus has been described before^59^. In brief, it expresses a p53 cDNA fused to the mVenus coding sequence under the control of the human EF1A promoter as well as a histone H2B cDNA fused to the mCerulean coding sequence under the control of the human Ubiquitin C promoter. We maintained these cells in McCoy’s 5 A (GE Healthcare Life Sciences, Freiburg, Germany) supplemented with 10% fetal calf serum (FCS; Thermo Fisher Scientific, Darmstadt, Germany) as well as penicillin and streptomycin. The selective antibiotics G418 (400 μg/ml, Carl Roth, Karlsruhe, Germany) and hygromycin (50 μg/ml, Thermo Fisher Scientific, Darmstadt, Germany) were added to maintain transgene expression.

The female human non-transformed breast epithelial cell line MCF10A expressing p53-mVenus and p21-mCherry has been described before^61^. In brief, the coding sequence for fluorescent proteins was inserted in the endogenous gene loci using Cas9-mediated genome engineering. The chromatin associated protein Cbx5 was tagged with mCerulean to serve as a nuclear marker. Cells were maintained in DMEM/F12 with 5% horse serum, 20 ng/ml EGF, 0.5 mg/ml hydrocortisone, 100 mg/ml cholera toxin and 10 mg/ml insulin. All media contained 2 mM Glutamax, 100 U/ml penicillin and 100 mg/ml streptomycin. All cell lines were cultures at 37 °C with 5% CO_2_ at saturated humidity if not indicated otherwise.

### Time‐lapse microscopy

We seeded 1 × 10^5^ cells in ibiTreat polymer-bottom plates (ibidi, Martinsried, Germany) 2 d before experiments. When indicated, cells where equilibrated at the given temperature for 14 h. The day of the experiment, medium was replaced by FluoroBrite medium (Thermo Fisher Scientific, Darmstadt, Germany) lacking phenol red and riboflavin. Cells were treated with X-ray irradiation at a dose rate of 1 Gy/26 s (250 KeV, 10 mA). We imaged cells on a Nikon Ti inverted fluorescence microscope (Nikon, Düsseldorf, Germany) with a Nikon DS‐Qi2 camera and a 20 × Plan Apo objective (numerical aperture 0.75) using appropriate filter sets (Venus: 500/20-nm excitation [EX], 515-nm dichroic beam splitter [BS], 535/30-nm emission [EM]; CFP: 436/20-nm EM, 455-nm BS, 480/40-nm EX). The microscope was enclosed with an incubation chamber set to temperatures in the range from 33 °C to 41 °C as indicated. The atmosphere was maintained at 5% CO_2_ concentration and saturated humidity. Cells were imaged every 15 min for the duration of the experiment using Nikon Elements software^59^.

### Image analysis and cell tracking

Cells were tracked throughout the duration of the experiment using custom‐written MATLAB (MathWorks) scripts based on code developed by the Alon laboratory^80^ and the CellProfiler project^81^. In brief, we applied flat field correction and background subtraction to raw images before segmenting individual nuclei from nuclear marker images using thresholding and seeded watershed algorithms. Segmented cells were then assigned to corresponding cells in following images using a greedy match algorithm. Only cells tracked from the first to last time point were considered. For the analyses we tracked cells in forward direction from the first to the last time point. Upon division, we followed the daughter cell closest to the last position of the mother and merged tracks from mothers and offspring. Using binary masks from nuclear marker images, we quantified mean nuclear fluorescence intensity in the p53/mVenus channel.

### Data pre-processing

Before analysis, we pre-processed our data with custom made filters to remove technical noise and signals from biological events unrelated to the induced response. To remove spikes and sustained intensity shifts caused by segmentation and tracking errors, we identified those features based on cell specific thresholds defined by the standard deviation of the processed trajectory and replaced them by linear interpolation. Spikes and sustained shifts were defined either by a decrease followed by an increase or an increase followed by a decrease both greater than the standard deviation of the processed trajectory. Removal of spikes and sustained shifts was done iteratively. This led to an increased signal-to-noise ratio due to local smoothing, emphasizing slower changing biological responses. As the nuclear envelop breaks down during mitosis and morphological changes lead to increased auto-fluorescence, we disregarded signals measured during cell division and removed them from the trajectories by interpolation. To identify cell division, we normalized for each cell the nuclear area and the integrated fluorescence intensity of the nuclear marker to their respective means, smoothed them by the approach described above and combined the two trajectories by averaging. We then applied a 1D Prewitt filter (length 75 minutes) to the combined trajectory to amplify signal discontinuities that correspond to cell divisions. Cell divisions events corresponded to a value of the Prewitt filtered trajectory exceeding a manually selected threshold based on several hundred visually detected cell divisions^82^.

### Normalization

Combining different time-lapse microscopy experiments is non-trivial due to multiple sources of variability that result in different distributions of the fluorescence intensity measured even among identical experimental settings. These comprise biological sources such as different passage numbers of cell lines uses as well as technical sources such as different light sources. To overcome the limitations of comparing different experiments, we propose a method that aims to reduce the inter-experiment deviation by fitting all experiments to one reference experiment. Our approach is based on the central assumption that we have a constant set of measures for all experiments with comparable measurement distributions. In this case, all deviations of the measure can be directly related to other sources of non-biological heterogeneity in data acquisition. In our experimental setup, we have for each experiment cells incubated at 37 °C with no radiation applied at the first time point, for which we assumed similar distributions. In the following example, we also assumed equal cell numbers in all experiments. This was not the case in our data sets, which required us to employ additional interpolation or extrapolation steps.

#### Example of normalization procedure

Let E_ijk_ be the set of our experimental measures, with *i* the experiment, *j* the time point and a cell *k*. We assume that all experiments have the same experimental condition at time point *j* = *1* and we set one experiment *x* as the reference. To fit another experiment y to the experiment x we use the following computation. The central part is the estimation of the coefficients *weight*. We start by computing the coefficient by *weight* = *sort(E(x,1,:))./sort(E(y,1,:))*. In the next steps we use the *weight* to fit the experiment *y* onto *x* by applying the coefficient to each time point.



The idea is that the non-biological error is constant over time and that we can estimate the error at the time point where we can assume identical conditions. In Supplement Fig. 1 we present some results of this normalization method. The presented data demonstrates that temporal dynamics and differences in the strength of the response are conserved after normalization among the different experimental conditions. This method gives us the opportunity to directly compare normalized measures of the abundance of p53 within the cell populations.

### Pitch detection - Average Magnitude Difference Function (AMDF)

Among the different pitch detection algorithms AMDF is the most commonly used. AMDF, a variation on autocorrelation analysis, was proposed by Ross *et al.* in 1974^62^ and is used for real time applications as it involves less computational effort^83^. We used window lengths between 4.5 h - 7 h and assumed a pitch period lower bound of 2 h. For robustness we computed the different pitch positions for the different window sizes and used the mean of overall window sizes for a certain pitch position.



### Feature detection

The aim of feature detection is the identification of patterns in time series data. In general, we aim to find pulses in our data. However, our approach is not limited in the kind of pattern we like to identify in the data, which can have any more or less complicated form.

The proposed method works in a two-step approach. First we normalize each trajectory using a band based on local minima and maxima (Suppl. Fig. 2B,C) followed by the detection itself that is based on a Smith-Waterman^65^ like version of the dynamic time warping^64^ approach. In the following we will describe both steps in detail.

The band normalization computes at the begin constants based on the time series data that are used for generating a band around each trajectory. These constants define different attributes of the bands like the width, a maximum value for the lower bound and a minimal level of the upper bound. Using these constants and anchors for the past and the future, we estimate for each trajectory a band, as shown in Suppl. Fig. 2B. The anchors are simple extensions of the trajectory. We than used the band to normalize the trajectory by subtracting for each time point the lower bound from the measured value and the upper bound and afterward divide the reduced value of the measure by the reduced value of the upper bound. This normalizes the trajectories to a fixed range between 0 and 1, (Suppl. Fig. 2C). The idea of band normalisation is to emphasise fluctuations at a longer temporal scale in the data, which is is necessary for the next step to work properly.

In the following feature detection step, we apply an adjusted dynamic time warping approach to find appearances of a symmetric peak pattern in the range 0–1 of length 4 h. In other applications of the method, this pattern can have a completely different shape appropriate for the specific demands. To find the defined pattern, first we initiated the scoring matrix as described in the literature^64^ except for the fact that the pattern can start and end at every position in the trajectory. This can simply be achieved by putting zeros at the first column of the scoring matrix and infinite into the upper row (assuming the columns correspond the values in the patter) at the initiation step. After filling the scoring matrix, we find the beginnings and ends of pattern occurrences by backtracking from the local minima in the last column. To improve the result, we afterwards filter overlapping occurrences.



Having the start and the end points of pulses we can straightforward extract several features (Suppl. Fig. 2A) of the different patterns. Suppl. Fig. 2D shows the identified pulses in a false colour representation sorted by the length of the time the first detected pulse ended.

### Image processing Fig. 5C,D

To enhance small pulses especially in the non-irradiated scenario, we have applied image processing techniques. First we sorted the trajectories according to the length of the first detected pulse after the temperature was reduced from the shortest to the longest. Then we applied a Gaussian filter (11 × 11) on the data matrix of the sorted time series data. On the filtered result we applied an additional median filter (31 × 31). We used the difference between the two filtered data matrices to enhance the pulses in the data as shown in Fig. 5C,D.

### Reverse transcriptase quantitative PCR

We extracted mRNA using High Pure RNA Isolation kits (Roche, Mannheim, Germany). cDNA was generated using ProtoScript reverse transcriptase (NEB, Ipswich, MA) and oligo-dT primers. Quantitative PCR was performed in triplicate using SYBR Green reagent (Roche, Basel) on a StepOnePlus PCR machine (Thermo Fisher Scientific, Darmstadt). Primer sequences were as follows: BAX forward, CTG ACG GCA ACT TCA ACT GG; BAX reverse, GAT CAG TTC CGG CAC CTT GG; GADD45 forward, GCA ATA TGA CTT TGG AGG AAT TCT C; GADD45 reverse, TGA CTC AGG GCT TTG CTG; XPC forward, GTC TCT ACA GCC AAT TCC TCT G; XPC reverse, CCT TTG CTG GTC TTT GGT TTG**;** p21 forward, TGG ACC TGT CAC TGT CTT GT and p21 reverse, TCC TGT GGG CGG ATT AG.

### Flow cytometry

Cells were plated in 6 cm dishes two days before experiments. Cells were exposed to 10 Gy X-ray radiation, harvested at indicated time points by trypsination, washed with 1xPBS, fixed with ice-cold 80% Ethanol/20% 1xPBS and stored at −20 °C until all samples were collected. For flow cytometry analysis, cells were washed with 1xPBS, stained with 25 mg/ml PI in 0.1% Triton 1xPBS with 0.2 mg/ml RNase A and analyzed on a Cytomics FC500 cytometer (Beckman Coulter). Cell cycle phases were determined based on the DNA content in FlowJo software (FlowJo, LLC).

## Supplementary information

Supplementary Movie S1.

Supplementary Movie S2.

Supplementary Movie S3.

Supplementary Movie S4.

Supplementary Movie S5.

Supplementary Movie S6.

Supplementary Movie S7.

Supplementary Movie S8.

Supplementary Movie S9.

Supplementary Movie S10.

Supplementary Informatiton.

## Data Availability

Matlab (Mathworks) was used to analyses and process data. Time series data of all tracked cells as well as data analysis scripts are available online (10.25534/tudatalib-34). Original image data and image analysis scripts are available from the corresponding author upon reasonable request.
