# Supplementary material for: p53 dynamics in single cells are temperature-sensitive
Source: Sci Rep. 2020 Jan 30;10:1481. doi: 10.1038/s41598-020-58267-1 (PMC6992775; doi:10.1038/s41598-020-58267-1)
Supplement: Supplementary file 11 — Supplementary Informatiton. [file 41598_2020_58267_MOESM11_ESM.pdf]

Supplementary Information

p53 dynamics in single cells are temperature-sensitive

Marcel Jentsch, Petra Snyder, Caibin Sheng, Elena Cristiano and Alexander Loewer[#]

Department of Biology, Technische Universität Darmstadt, Darmstadt, Germany

Supplementary Figure 1: Normalization 2
Supplementary Figure 2: Statistical analysis of time-lapse data 3
Supplementary Figure 3: MCF10A cells 4
Supplementary Figure 4: Auto-correlation 5
Supplementary Figure 5: Features 6
Supplementary Figure 6: Gene expression and cellular outcome 7
Supplementary Table 1: Number of analysed cells per conditions 8

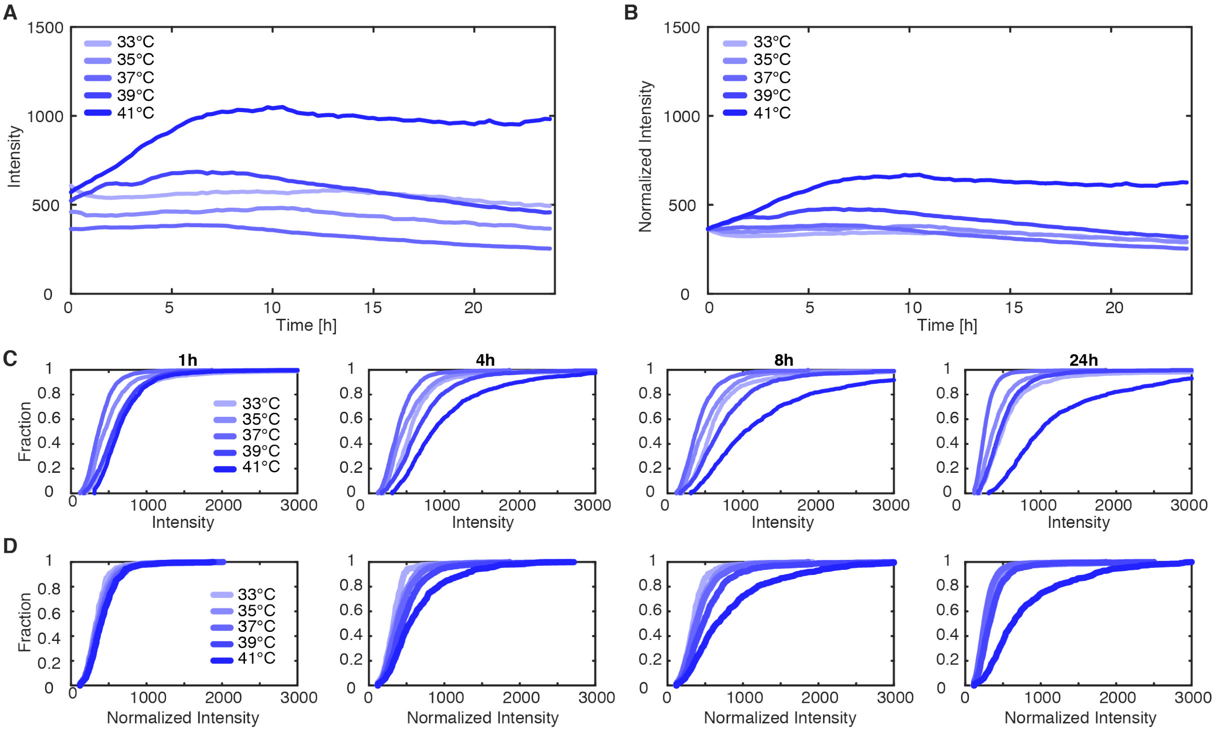

Supplementary Figure 1: Normalization

(A) Median raw fluorescence intensities of p53-Venus for non-irradiated cells at different temperatures.

(B) Median normalized fluorescence intensities of p53-Venus for non-irradiated cells at different temperatures.

(C-D) Cumulative frequency distribution of raw (C) and normalized (D) fluorescence intensities of p53-Venus for non-irradiated cells at different temperatures and indicated time points. Deviations related to temperature remain conserved while differences among experiments are drastically reduced.

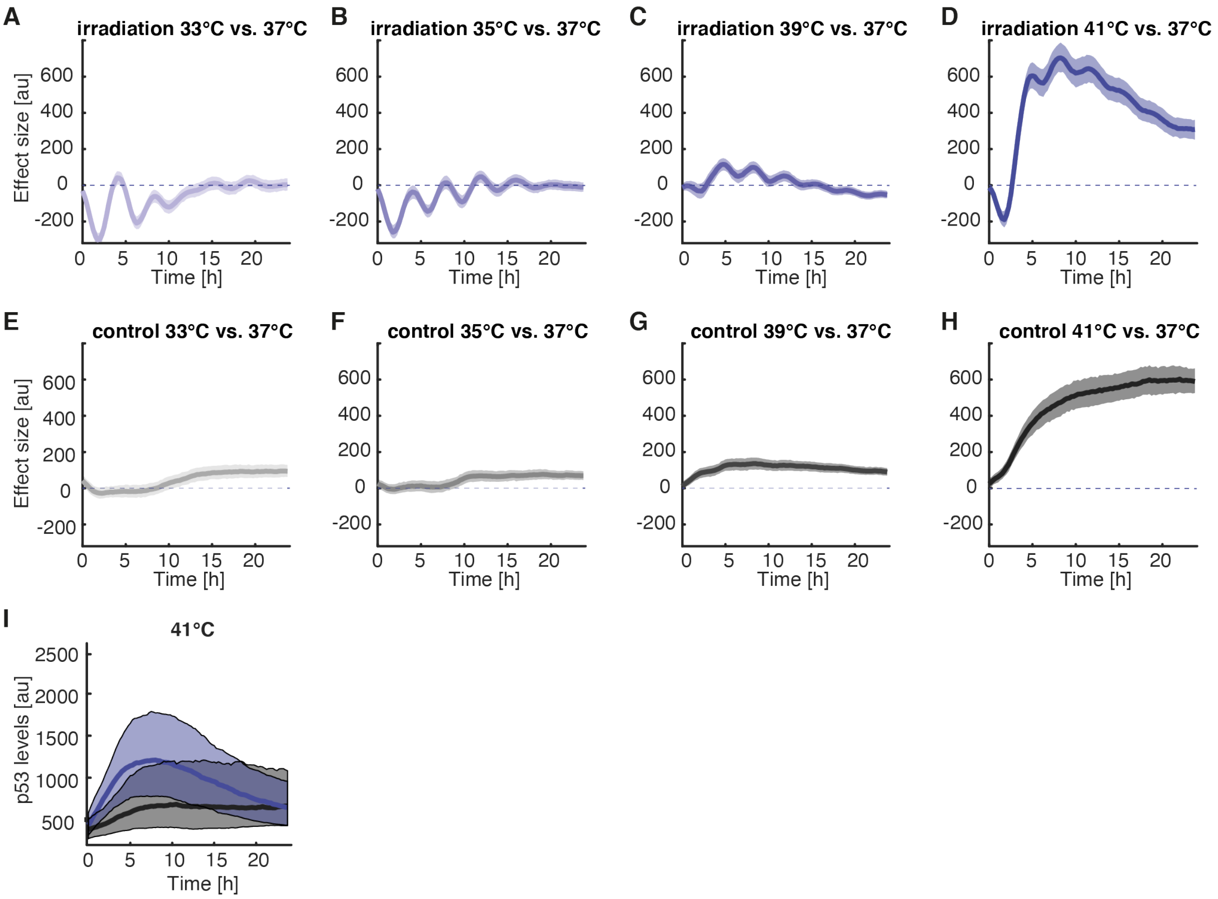

Supplementary Figure 2: Statistical analysis of time-lapse data

(A-H) Estimated mean differences between irradiated (A-D) and non-irradiated cells (E-H) incubated at the indicated temperatures compared to cells incubated at 37°C (solid lines); shaded areas indicate 95% confidence intervals determined by permutation testing. Dashed lines serve as guides to the eye. Compare to Figure 2A-E.

(I) Population level p53 responses of cells pre-equilibrated at 41°C. Data from Figure 2E shown with different scale of the y-axis for full representation (black: non-irradiated, blue: 10Gy IR). Bold lines indicate median p53 levels, shaded areas reflect the inter-quantile ranges from 0.25 to 0.75.

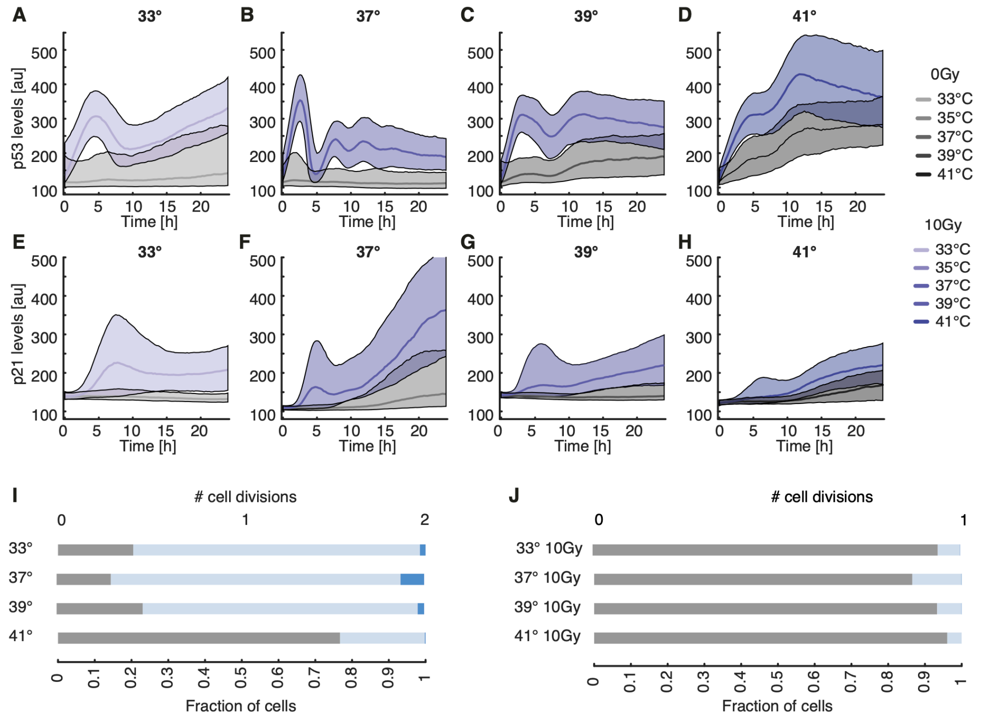

Supplementary Figure 3: MCF10A cells

(A-D) Population level p53 responses of pre-equilibrated non-transformed breast-epithelial MCF10A cells for the temperature range from 33°C to 41°C (black: non-irradiated, blue: 10Gy IR). Bold lines indicate median p53 levels, shaded areas reflect the inter-quantile ranges from 0.25 to 0.75. See Table S1 for number of analysed cells.

(E-H) Population level p21 responses of pre-equilibrated non-transformed breast-epithelial MCF10A cells for the temperature range from 33°C to 41°C (black: non-irradiated, blue: 10Gy IR). Bold lines indicate median p53 levels, shaded areas reflect the inter-quantile ranges from 0.25 to 0.75. See Table S1 for number of analysed cells.

(I-J) Cell proliferation shown as number of cell divisions per cell within 24 h for non-irradiated (I) and irradiated (J) MCF10A cells incubated at the indicated temperatures.

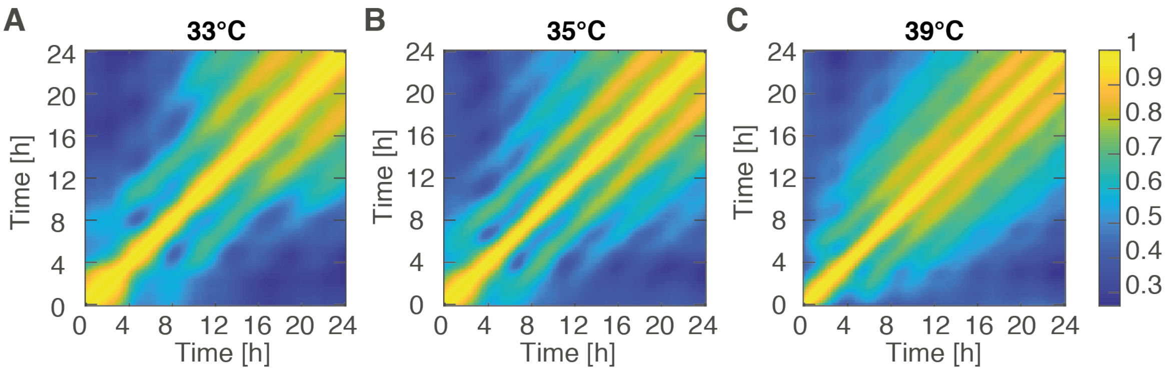

Supplementary Figure 4: Auto-correlation

(A-C) Auto-correlation among irradiated cells at indicated temperatures. Each point in this graph represents the correlation coefficient between p53 levels in the population at two different time points, except along the main-diagonal. 37°C and 41°C are shown in Figure 3.

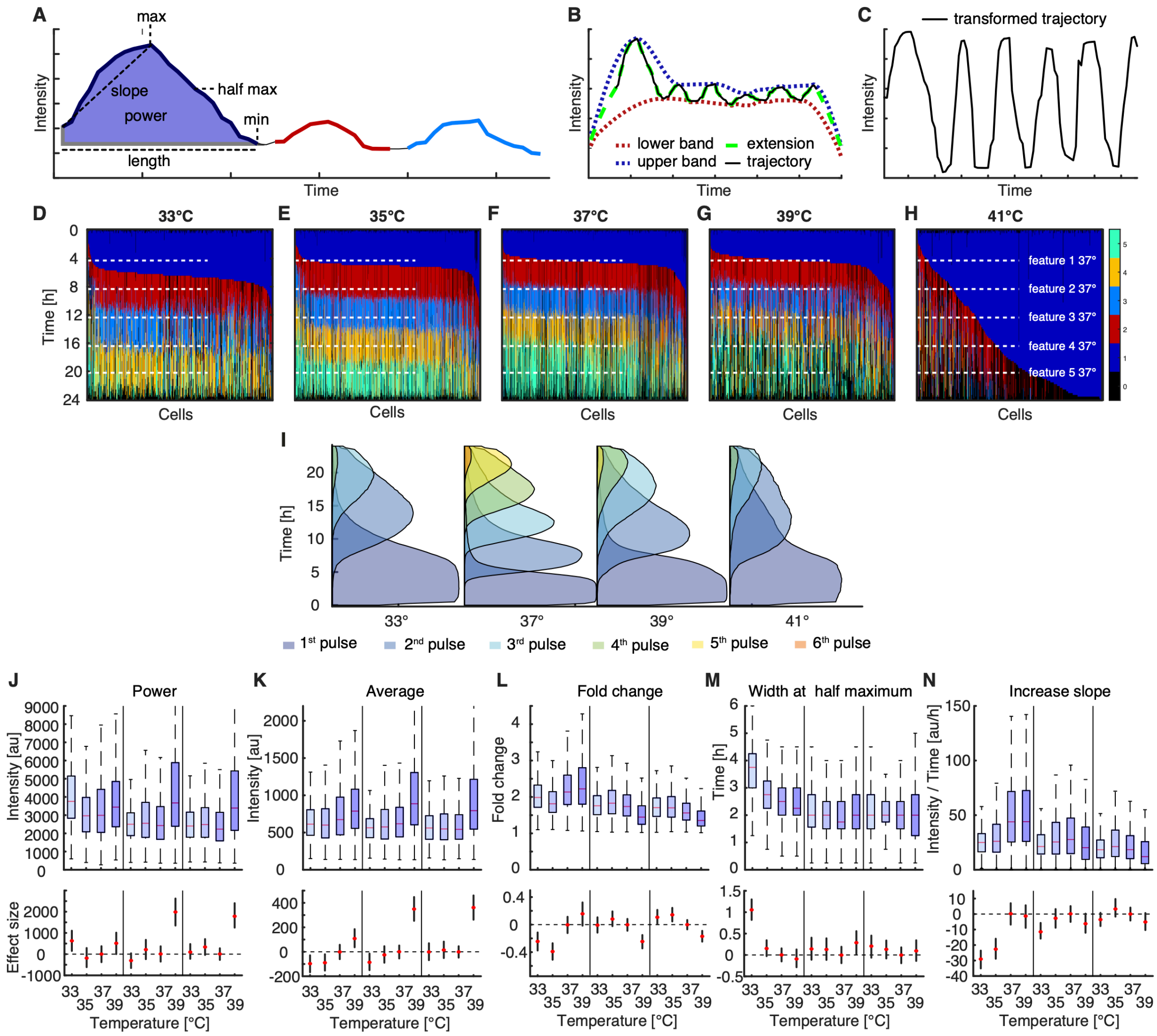

Supplementary Figure 5: Features

(A) Example of a trajectory with detected features/pulses and schematic representation of the readouts of feature characteristics.
(B) Our dynamic time warping based feature detection method generates initially a band around the input trajectory that is used to compensate local amplitude effects.
(C) The upper and the lower bound of the band generated in (B) is used to scale the input trajectory locally in the range of 0 to 1.
(D-H) False colour heat maps of the detected features upon damage induction for the temperature range from 33°C to 41°C. The data shown corresponds to the data shown in Figure 3. Each colour corresponds to the temporal order of the detected features.
(I) Probabilities of being in a certain pulse state at a given time point for irradiated MCF10A cells (10Gy) over a temperature range from 33°C to 41°C. Pulses are detected using a local dynamic time warping based approach. The pulsing frequency is lower at 33°C compared to 37°C. At temperature above 37°C, pulsing dynamics are less pronounced and increasingly replaced by a sustained response.
(I-M) Additional features for the first three detected pulses. The data shown corresponds to the data sets shown in Figure 3. A scheme of the readouts is shown in panel (A). Estimated changes compared to cells incubated at 37°C are indicated below (red dots); error bars represent 95% confidence intervals determined by permutation testing. Dashed lines serve as guides to the eye.

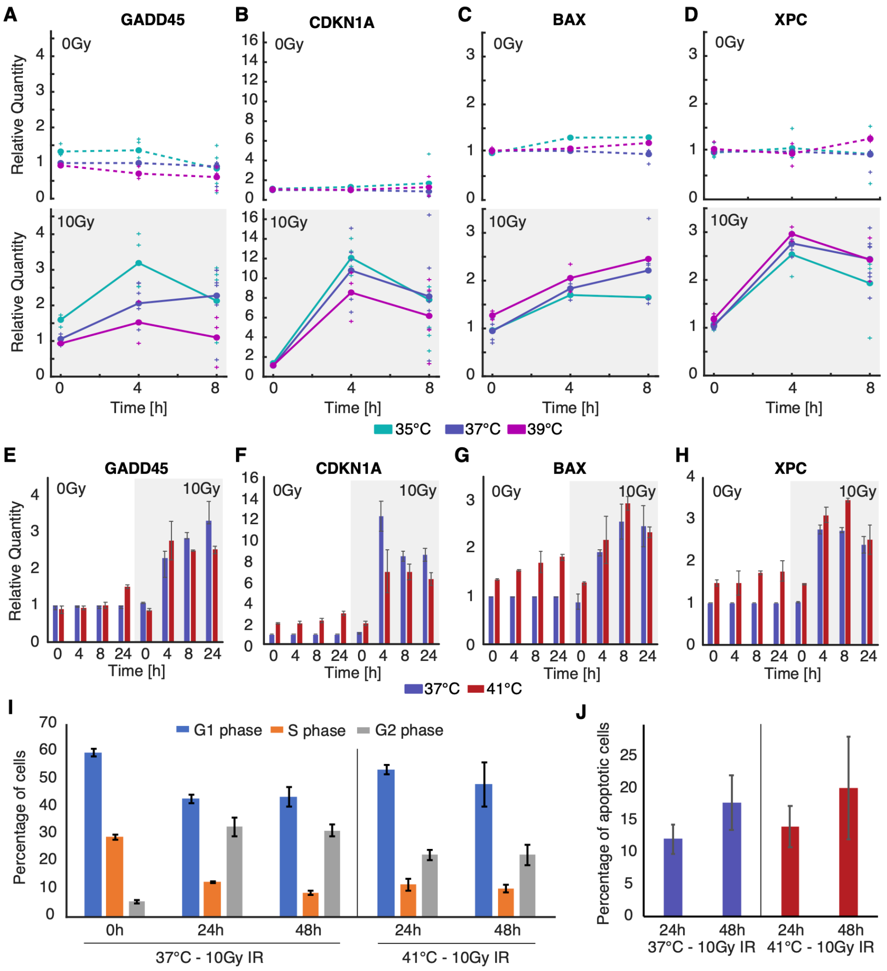

Supplementary Figure 6: Gene expression and cellular outcome

(A-D) Induction of mRNA expression of p53 target genes CDKN1A/p21, GADD45 (both cell cycle arrest), XPC (DNA damage repair) and BAX (apoptosis) were measured in non-irradiated cells (upper row) and upon irradiation with 10Gy IR (lower row) at indicated time points and temperatures by RT-qPCR. β-Actin was used as an internal control. Mean induction levels from 3-6 biological repeats are shown as dots, individual measurements as crosses. Lines serve as guides to the eye.

(E-H) Induction of target gene expression was measured in non-irradiated and irradiated cells (10Gy) at 37°C and 41°C at the indicated time points. Mean induction levels from three biological repeats are shown, error bars represent standard error of the mean.

(I) Cell cycle distributions were measured by flow cytometry at the indicated time points after treatment with ionizing radiation (10Gy) at 37°C and 41°C. Error bars indicate standard deviation of triplicates.

(J) Percentages of apoptotic cells (subG1 fraction) was measured by flow cytometry 24h and 48h after irradiation (10Gy) at 37°C and 41°C. Error bars indicate standard deviation of triplicates.

Condition	Number of cells	Figure
Non-irradiated A549 cells equilibrated to 37°C	2857	Figure 1E
Irradiated A549 cells (10Gy IR) equilibrated to 37°C	1182	Figure 1E
Non-irradiated A549 cells equilibrated to 33°C	1214	Figure 2A
Irradiated A549 cells (10Gy IR) equilibrated to 33°C	782	Figure 2A
Non-irradiated A549 cells equilibrated to 35°C	1759	Figure 2B
Irradiated A549 cells (10Gy IR) equilibrated to 35°C	1098	Figure 2B
Non-irradiated A549 cells equilibrated to 37°C	3830	Figure 2C
Irradiated A549 cells (10Gy IR) equilibrated to 37°C	2857	Figure 2C
Non-irradiated A549 cells equilibrated to 39°C	2436	Figure 2D
Irradiated A549 cells (10Gy IR) equilibrated to 39°C	1630	Figure 2D
Non-irradiated A549 cells equilibrated to 41°C	1139	Figure 2E
Irradiated A549 cells (10Gy IR) equilibrated to 41°C	1007	Figure 2E
Non-irradiated A549 cells acutely shifted to 41°C	721	Figure 2H
Irradiated A549 cells (10Gy IR) acutely shifted to 41°C	994	Figure 2H
Irradiated A549 cells (10Gy IR) damaged at 37°C	1420	Figure 4A
Irradiated A549 cells (10Gy IR) damaged at 37°C and shifted to 33°C after 6h	1407	Figure 4A
Irradiated A549 cells (10Gy IR) damaged at 37°C and shifted to 35°C after 6h	1488	Figure 4A
Irradiated A549 cells (10Gy IR) damaged at 37°C and shifted to 39°C after 6h	935	Figure 4A
Irradiated A549 cells (10Gy IR) damaged at 37°C and shifted to 41°C after 6h	909	Figure 4A
Non-irradiated A549 cells incubated at 41°C and shifted to 37°C after 6h	449	Figure 5A
Irradiated A549 cells (10Gy IR) damaged at 41°C and shifted to 37°C after 6h	2213	Figure 5A
Non-irradiated A549 cells incubated at 37°C	519	Figure 5A
Irradiated A549 cells (10Gy IR) equilibrated to 33°C	2519	Figure S3 A,E
Non-irradiated A549 cells equilibrated to 33°C	2493	Figure S3 A,E
Irradiated A549 cells (10Gy IR) equilibrated to 37°C	3134	Figure S3 B,F
Non-irradiated A549 cells equilibrated to 37°C	3018	Figure S3 B,F
Irradiated A549 cells (10Gy IR) equilibrated to 39°C	3650	Figure S3 C,G
Non-irradiated A549 cells equilibrated to 39°C	3237	Figure S3 C,G
Irradiated A549 cells (10Gy IR) equilibrated to 41°C	681	Figure S3 D,H
Non-irradiated A549 cells equilibrated to 41°C	307	Figure S3 D,H

Supplementary Table 1: Number of analysed cells per conditions
